# Tuberculosis non-communicable disease comorbidity and multimorbidity in public primary care patients in South Africa

**DOI:** 10.4102/phcfm.v10i1.1651

**Published:** 2018-04-11

**Authors:** Karl Peltzer

**Affiliations:** 1 HIV/STI and TB (HAST) Research Programme, Human Sciences Research Council, Pretoria, South Africa; 2 Department of Psychology, University of Limpopo, Turfloop, South Africa

## Abstract

**Background:**

Little is known about the prevalence of non-communicable disease (NCD) multimorbidity among tuberculosis (TB) patients in Africa.

**Aim and setting:**

The aim of this study was to assess the prevalence of NCD multimorbidity, its pattern and impact on adverse health outcomes among patients with TB in public primary care in three selected districts of South Africa.

**Methods:**

In a cross-sectional survey, new TB and TB retreatment patients were interviewed, and medical records assessed in consecutive sampling within 1 month of anti-TB treatment. The sample included 4207 (54.5% men and 45.5% women) TB patients from 42 primary care clinics in three districts. Multimorbidity was measured as the simultaneous presence of two or more of 10 chronic conditions, including myocardial infarction or angina pectoris, arthritis, asthma, chronic lung disease, diabetes mellitus, hypertension, dyslipidaemia, malignant neoplasms, tobacco and alcohol-use disorder.

**Results:**

The prevalence of comorbidity (with one NCD) was 26.9% and multimorbidity (with two or more NCDs) was 25.3%. We identified three patterns of multimorbidity: (1) cardio-metabolic disorders; (2) respiratory disorders, arthritis and cancer; and (3) substance-use disorders. The likelihood of multimorbidity was higher in older age, among men, and was lower in those with higher education and socio-economic status. The prevalence of physical health decreased, and common mental disorders and post-traumatic stress disorder increased with an increase in the number of chronic conditions.

**Conclusion:**

High NCD comorbidity and multimorbidity were found among TB patients predicted by socio-economic disparity.

## Introduction

The global burden of non-communicable diseases (NCDs), in particular cancer, cardiovascular disease, diabetes and chronic respiratory disease, accounts for two-thirds of mortality worldwide,^1^ and the fastest increase in NCDs has been recorded in low- and middle-income countries, particularly in sub-Saharan Africa.^2^ This rapid increase of NCDs may be because of an ageing population, rapid urbanisation, changes in environmental factors and lifestyle changes.^3,4^ South Africa is confronted with a large burden of disease from both chronic infections and NCDs.^5^ ‘People living with chronic communicable diseases such as tuberculosis (TB) and human immunodeficiency virus (HIV) are most likely to develop comorbidity with NCDs’.^6^ With the increase of chronic communicable and NCDs, primary health care services are confronted with a huge challenge.^5^

In a study among NCD (hypertension, diabetes, chronic respiratory disease and depression) primary care patients in South Africa, considerable multimorbidity was found and unmet treatment needs existed (4% in hypertension group received no treatment or medicines, 6.5% in the diabetes group, 49.0% in the respiratory diseases group and 77.1% in the depression group).^7^ In another primary care study in Cape Town, South Africa, based on the analysis of treatment prescriptions, a high comorbid diseases pattern was found for TB patients, with 80% having HIV, 37% hypertension and 12% diabetes mellitus.^8^ In a study on NCD multimorbidity (arthritis, cancer, diabetes mellitus, hypertension, heart disease, obstructive lung disease and psychiatric problems), based on medical record review, 1.14% among a large sample of TB patients in Brazil had multimorbidity,^9^ with the highest prevalence of coexisting diabetes mellitus, followed by hypertension, psychiatric disease, cardiovascular disease, cancer, chronic pulmonary obstructive disease and arthritis.^9^

Active TB disease is linked with the breakdown in immune surveillance.^10^ This explicates the strong association between active TB disease and other communicable diseases or NCDs that exercise a toll on the immune system.^10^ Common TB-NCD comorbidities include diabetes mellitus, smoking, alcohol-use disorders, chronic lung diseases, cancer and depression.^6,11,12^ For example, globally, the overall median prevalence of diabetes mellitus among TB patients was 16% [interquartile range (IQR) 9.0% – 25.3%].^13^ Factors associated with TB-NCD multimorbidity may include older age,^9,14^ female gender^9,14,15^ and lower socio-economic status,^14^ and an insignificant association was found for HIV-positive status.^9^ Moreover, TB-NCD multimorbidity has been found to have negative effects on physical and mental health and quality of life.^16,17,18^

Estimates of TB-NCD multimorbidity, its pattern and health impact may better inform health care providers in integrating NCD management into TB care.^9^ In response to the dual burden of HIV and AIDS and NCDs, the integrated chronic disease management model was introduced in South Africa.^19^ The aim of this study was to assess the prevalence of NCD multimorbidity and its pattern on adverse health outcomes among public primary care patients with TB in three selected districts of South Africa.

## Research methods and design

### Sample and procedure

In a cross-sectional survey, 4207 new TB and retreatment patients aged ≥ 18 years were consecutively recruited within 1 month of anti-TB treatment being initiated from 42 high TB caseloads primary health care facilities in three high TB caseloads districts (Siyanda, Nelson Mandela Metro and eThekwini) in South Africa. Participants were interviewed with a structured (close-ended) questionnaire, and medical records were accessed by external research assistants from May to October in 2011.^20^ Written informed consent was obtained from all participants prior to the study.

### Measures

Tuberculosis patients were asked about NCDs:

Has a doctor or nurse or health worker at a clinic or hospital ever told you that you have or have had any of the following conditions: Hypertension/high blood pressure? Heart attack or angina (chest pains)? High blood cholesterol or fats in the blood? Diabetes or blood sugar? Chronic lung disease such as emphysema and bronchitis? Asthma? Sore joints, e.g. arthritis, gout? Cancer or a malignancy of any kind? Tobacco use problem? and Alcohol use problem?^21^

Those who responded with ‘yes’ were asked, ‘Are you currently taking medicines/treatment for this disease?’ (Response options ‘yes or no’).^21^

In addition, alcohol and tobacco use were assessed with structured questionnaires. The 10-item Alcohol Use Disorder Identification Test (AUDIT)^22^ assessed alcohol consumption level (3 items), symptoms of alcohol dependence (3 items) and problems associated with alcohol use (4 items) (Cronbach’s alpha 0.92).^20^ Alcohol-use disorder was defined as 8 or more scores for men and 7 or more scores for women on the AUDIT (range 0–40). For assessing tobacco use, two questions were asked about the use of tobacco products: (1) ‘Do you currently use one or more of the following tobacco products (cigarettes, snuff, chewing tobacco, cigars, etc.)?’ Response options were ‘yes’ or ‘no’. (2) ‘In the past month, how often have you used one or more of the following tobacco products (cigarettes, snuff, chewing tobacco, cigars, etc.)?’ Response options were once or twice, weekly, almost daily and daily.^23^

Moreover, the history of a ‘sexually transmitted infection (STI), for example herpes, gonorrhoea, chlamydia and genital warts’, was assessed by self-report; HIV status and TB treatment status were assessed by medical record review.

General physical and mental health were assessed with the social functioning (SF)-12^24^ (Cronbach’s alpha 0.80). For each respondent, the SF-12 scoring algorithm generates a physical health component summary (PCS-12) score and a mental health component summary (MCS-12) score. The lower the physical health score (PCS-12) or mental health score (MCS-12), the more activity limitations a person has.^24^

The Kessler Psychological Distress Scale (K-10) was used to measure anxiety or depressive disorders^25^ (Cronbach’s alpha 0.92). A screening cut-off score of 28 was used to best define depressive and anxiety disorders.^26^

The 4-item Primary Care Post-Traumatic Stress Disorder (PC-PTSD) Screen was used to assess PTSD symptoms^27^ (Cronbach’s alpha 0.89). A cut-off of 2 was used for PTSD-positive screening.^27^

Socio-demographic questions included age, gender, education, marital and residence status. Poverty was assessed with five items on the availability or non-availability of shelter, fuel or electricity, clean water, food and cash income in the past week. Response options ranged from 1 = ‘not one day’ to 4 = ‘every day of the week’^28^ (Cronbach’s alpha 0.89). Poverty was defined as higher scores on non-availability of essential items.

### Data analysis

Data were analysed using the IBM Statistical Package for the Social Sciences (SPSS) for Windows version 24.0 (SPSS, Inc., Chicago, IL, USA). Frequencies, means and standard deviations were computed to describe the sample. Data were checked for normality distribution and outliers. Chi-square tests were used to test for differences in proportions. To identify the pattern of NCD multimorbidity, principal factor method was used with equimax rotation. Multinomial logistic regression was used by comparing NCD comorbidity and NCD multimorbidity prevalence with those not having any NCD (reference category). Linear regression was used to predict general physical and mental health, and logistic regression was used to predict psychological distress and PTSD symptoms. Probability below 0.05 was regarded as statistically significant.

### Ethical consideration

The study protocol was approved by the Human Sciences Research Council Research Ethics Committee (Protocol REC No.1/16/02/11). The Department of Health in South Africa has also provided approval for this study.

## Results

### Sample and multimorbidity

The sample included 4207 public primary care TB patients, 53.5% male patients and 46.5% female patients, mean age 36.2 years (s.d. = 11.4), age range 18–93 years. The prevalence of individual NCDs, in order of frequency, included alcohol-use disorder (24.3%), daily tobacco use (15.0%), hypertension (8.9%), ischaemic heart disease or angina (7.5%), arthritis (4.5%), type 2 diabetes (4.1%), asthma (3.5%), cancer or malignant neoplasms (2.1%), chronic lung disease (1.9%) and dyslipidaemia (1.6%). The overall comorbidity (with one NCD) was 26.9% and multimorbidity (with two or more NCDs) was 20.9%. There was a large unmet need for treatment or medication, ranging from 25.0% for ischaemic heart disease or angina to 97.7% for alcohol-use disorders (Table 1).

**TABLE 1:** Prevalence of individual non-communicable diseases, comorbidity and multimorbidity stratified by gender.

NCD	All *N* (%)	Proportion currently taking treatment (%)	Male *N*† (%)	Female *N*† (%)	*p*
Hypertension	373 (8.9)	72.6	163 (7.4)	206 (10.7)	< 0.001
Ischaemic heart disease or angina pectoris	316 (7.5)	75.0	168 (7.6)	142 (7.4)	0.796
Type 2 diabetes	171 (4.1)	66.1	76 (3.4)	89 (4.6)	0.051
Dyslipidaemia	69 (1.6)	60.0	33 (1.5)	33 (1.7)	0.567
Chronic lung disease	79 (1.9)	62.0	42 (1.9)	37 (1.9)	0.954
Cancer or malignant neoplasms	88 (2.1)	47.8	39 (1.8)	48 (2.5)	0.102
Arthritis	190 (4.5)	51.0	92 (4.2)	95 (4.9)	0.229
Asthma	149 (3.5)	61.7	72 (3.2)	73 (3.8)	0.346
Daily tobacco use	632 (15.0)	4.3	500 (22.6)	122 (6.3)	< 0.001
Alcohol-use disorder	1022 (24.3)	2.3	745 (33.6)	260 (13.5)	< 0.001
Comorbidity	1030 (26.9)	-	680 (30.7)	435 (22.6)	< 0.001
Multimorbidity	879 (20.9)	-	580 (26.2)	284 (14.7)	< 0.001

NCD, non-communicable disease.

† , Because of missing values, numbers do not always add up.

### Patterns of non-communicable disease multimorbidity

In the factor analysis, three factors emerged with a total of 42.3 cumulative per cent. The Kaiser–Meyer–Orkin measure of 0.69 indicated a moderate sampling adequacy. The first factor (eigenvalue = 1.82) was characterised by high loadings for hypertension (0.73), diabetes (0.68), dyslipidaemia (0.50) and ischaemic heart disease or angina (0.44), named cardiovascular-metabolic disorders. The second factor (eigenvalue = 1.33) was characterised by moderately high loadings of asthma (0.61), arthritis (0.61), cancer (0.60) and chronic lung diseases (0.46), named respiratory diseases, arthritis and cancer. The third factor (eigenvalue = 1.10) had high loadings on daily or almost daily tobacco use (0.81) and alcohol-use disorders (0.81), named substance-use disorders.

The distribution of comorbidity and multimorbidity is shown in Tables 2 and 3. Compared with TB patients without NCDs, those with one (comorbidity) NCD and those with two or more (multimorbidity) NCDs were likely to be older, men, married or separated or widowed; had lower education and greater poverty; were HIV negative; and had had an STI (other than HIV). In addition, being a TB retreatment patient was associated with NCD multimorbidity (Table 3).

**TABLE 2:** Prevalence of non-communicable disease comorbidity and multimorbidity stratified by patient characteristics.

Variable (*N*, %)	Comorbidity prevalence *N*† (%)	Multimorbidity prevalence *N*† (%)
**Age in years** 18–30 (1560, 37.6) 31–44 (1719, 41.4) 45 or more (869, 20.9)	389 (24.9) 459 (26.7) 262 (30.1)	224 (14.4) 331 (19.3) 311 (35.8)
**Gender** Female (1927, 46.5) Male (2216, 53.5)	435 (22.6) 680 (30.7)	284 (14.7) 580 (26.2)
**Marital status** Unmarried (2885, 71.8) Married/cohabitating (887, 22.1) Separated/divorced/widowed (244, 6.1)	734 (25.4) 263 (29.7) 84 (34.4)	484 (16.8) 270 (30.4) 81 (33.2)
**Education** Grade 7 or less (1086, 26.1) Grade 8–11 (1932, 46.5) Grade 12 or more (1139, 27.4)	318 (29.3) 536 (27.7) 260 (22.8)	340 (31.3) 396 (20.5) 134 (11.8)
**Poverty** Low (1452, 36.3) Medium (2000, 50.0) High (550, 13.7)	356 (24.5) 565 (28.3) 155 (28.2)	255 (17.6) 414 (20.7) 155 (28.2)
**Residence** Rural (828, 19.9) Urban (3339, 80.1)	229 (27.7) 887 (26.6)	148 (17.9) 728 (21.8)
**TB treatment status** New TB patient (3183, 76.3) TB retreatment patient (991, 23.7)	862 (27.1) 257 (25.9)	601 (18.9) 273 (27.5)
**HIV status** Negative or unknown status (1794, 43.7) Positive (2315, 56.3)	524 (29.2) 581 (25.1)	441 (24.6) 419 (18.1)
**Sexually transmitted infection** No (3923, 93.5) Yes (273, 6.5)	1043 (26.6) 86 (31.5)	762 (19.4) 114 (41.8)

TB, tuberculosis; HIV, human immunodeficiency virus.

† , because of missing values, numbers do not always add up.

**TABLE 3:** Multinomial logistic regression model examining correlates of multimorbidity.

Variable	One NCD versus none AOR (95% CI)	Multimorbidity versus no NCD AOR (95% CI)
**Age in years** 18–30 31–44 45 or more	1.00 (Reference) 1.01 (0.84, 1.24) 1.48 (1.15, 1.91)**	1.00 (Reference) 1.14 (0.91, 1.43) 2.48 (1.88, 3.28)***
**Gender** Female Male	1.00 (Reference) 1.96 (1.66, 2.31)***	1.00 (Reference) 2.34 (1.93, 2.84)***
**Marital status** Unmarried Married/cohabitating Separated/divorced/widowed	1.00 (Reference) 1.30 (1.05, 1.60)* 1.67 (1.16, 2.39)**	1.00 (Reference) 1.76 (1.40, 2.20)*** 1.88 (1.29, 2.74)***
**Education** Grade 7 or less Grade 8–11 Grade 12 or more	1.00 (Reference) 0.88 (0.72, 1.09) 0.65 (0.51, 0.83)***	1.00 (Reference) 0.68 (0.54, 0.85)*** 0.40 (0.30, 0.53)***
**Poverty** Low Medium High	1.00 (Reference) 1.27 (1.06, 1.51)** 1.40 (1.07, 1.83)*	1.00 (Reference) 1.12 (0.91, 1.38) 1.81 (1.36, 2.41)***
**Residence** Rural Urban	1.00 (Reference) 1.02 (0.84, 1.25)	1.00 (Reference) 1.26 (0.99, 1.61)
**TB treatment status** New TB patient TB retreatment patient	1.00 (Reference) 1.02 (0.84, 1.24)	1.00 (Reference) 1.61 (1.31, 1.98)***
**HIV status** Negative or unknown status Positive	1.00 (Reference) 0.76 (0.64, 0.90)***	1.00 (Reference) 0.64 (0.53, 0.78)***
**Sexually transmitted infection** No Yes	1.00 (Reference) 2.04 (1.35, 3.06)***	1.00 (Reference) 4.49 (3.02, 6.70)***

AOR, adjusted odds ratio; CI, confidence interval; NCD, non-communicable disease; TB, tuberculosis; HIV, human immunodeficiency virus.

*** , *p* < 0.001;

** , *p* < 0.01;

* , *p* < 0.05.

### Association of non-communicable disease multimorbidity with four health outcome measures

The effects of multimorbidity on general physical health, general mental health, psychological distress and PTSD symptoms are shown in Table 4. In the unadjusted and adjusted models, having three of more NCDs was negatively associated with general physical health. As the number of NCDs increased, PTSD symptoms increased in both unadjusted and adjusted models. The number of NCDs was in the unadjusted model and in the adjusted model with socio-demographic factors associated with psychological distress, but when adjusted with HIV-coinfection psychological distress became non-significant. No association was found between NCD multimorbidity and general mental health (Table 4).

**TABLE 4:** Multimorbidity and physical and mental health outcomes.

Variables	PCS B (95% CI)	MCS B (95% CI)	Psychological distress (28 or more) (30.5%) OR (95% CI)	PTSD (26.1%) OR (95% CI)
**Model 1: Unadjusted**				
0 NCD 1 NCD 2 NCDs 3 or more NCDs	Reference −0.22 (−0.92 to 0.47) −0.62 (−1.48 to 0.24) −1.56 (−2.86 to −0.27)*	Reference −0.10 (−0.72 to 0.92) −0.51 (−1.52 to 0.51) −0.28 (−1.80 to 1.25)	1 (Reference) 1.02 (0.87, 1.19) 1.29 (1.06, 1.55)** 1.51 (1.16, 1.98)**	1 (Reference) 1.26 (1.07, 1.48)** 1.58 (1.30, 1.92)** 1.40 (1.05, 1.86)*
**Model 2: Adjusted for age, sex, education, income and residence**				
0 NCD 1 NCD 2 NCDS 3 or more NCDs	Reference −0.06 (−0.78 to 0.66) −0.63 (−1.53 to 0.27) −1.65 (−3.01 to −0.30)*	Reference 0.39 (−0.45 to 1.24) −0.08 (−1.13 to 0.97) 0.41 (−1.18 to 2.00)	(Reference) 0.94 (0.79, 1.11) 1.20 (0.98, 1.47) 1.35 (1.02, 1.78)*	1 (Reference) 1.36 (1.14, 1.62)*** 1.76 (1.42, 2.17)*** 1.63 (1.21, 2.21)**
**Model 3: Adjusted for SES and HIV co-infection**				
0 NCD 1 NCD 2 NCDS 3 or more NCDs	Reference −0.03 (−0.77 to 0.72) −0.72 (−1.54 to 0.21) −2.08 (−3.48 to −0.68)**	Reference 0.70 (−0.19 to 1.58) 0.30 (−0.71 to 1.50) 0.93 (−0.73 to 2.60)	1 (Reference) 0.95 (0.80, 1.13) 1.22 (0.99, 1.50) 1.34 (0.99, 1.79)	1 (Reference) 1.42 (1.18, 1.69)*** 1.79 (1.44, 2.22)*** 1.77 (1.30, 2.41)***

*** , *p* < 0.001;

** , *p* < 0.01;

* , *p* < 0.05.

PCS, physical health component summary; MCS, mental health component summary; PTSD, post-traumatic stress disorder; CI, confidence interval; OR, odds ratio; NCD, non-communicable disease; SES, socio-economic status.

## Discussion

The study, probably the first study in Africa, found a high prevalence of TB-NCD comorbidity (26.9%) and TB-NCD multimorbidity (25.3%), much higher than in a study in Brazil with 1.14% TB-NCD multimorbidity.^9^ One explanation for the low multimorbidity prevalence in the Brazil study may be the use of health records as recruitment strategy, because the latter may under report diseases.^14^ The findings of this study may be similar to a primary care study in South Africa, including the chronic conditions of HIV, TB, diabetes and hypertension, showing a multimorbidity of 22.6%.^8^ In comparison, the multimorbidity in NCD primary care patients in South Africa was 14.4%.^29^ In agreement with the Brazil study,^9^ this study found that the most common comorbid NCDs included mental or substance-use disorders, hypertension, cardiovascular disorders and type 2 diabetes. As found in a previous study among NCD primary care patients in South Africa,^7^ this study found that significant unmet treatment needs existed, ranging from 25.0% for ischaemic heart disease or angina, 27.4% for hypertension, 33.9% for type 2 diabetes to 97.7% for alcohol-use disorders.

The study identified three patterns of TB-NCD multimorbidity: (1) cardio-metabolic disorders; (2) respiratory disorders, arthritis and cancer; and (3) substance-use disorders. A similar three-factor multimorbidity pattern was observed in a systematic review:^30^ a group that included cardio-metabolic diseases; a second group that included at least one musculoskeletal disorder, including arthritis; and a third group that included at least one mental or substance disorder. Violan et al.^14^ also found in a review that most frequent comorbidities included arthritis and a cardio-metabolic cluster, including high blood pressure, diabetes and ischaemic heart disease. The prevalence of specific comorbid chronic conditions among TB patients in this study seemed to have been lower (56.3% for HIV, hypertension 8.9%, type 2 diabetes 4.1% and dyslipidaemia 1.6%) than in a primary care study in Cape Town, South Africa, based on the analysis of treatment prescriptions, with 80% having HIV, 37% hypertension and 12% diabetes mellitus,^8^ and in the case of self-reported hypertension (16.3%). But the found prevalence was similar to measured hypertension (10.2%) and self-reported dyslipidaemia (4.2%) and measured dyslipidaemia (19.2%) among the general adult population in South Africa.^30^ It was also similar to self-reported diabetes (5%) but lower than measured diabetes (9.6%) in the general adult population in South Africa.^31^

This study found, in agreement with previous studies,^9^ that older TB patients were more likely to have TB-NCD multimorbidity than younger TB patients, which is consistent with the idea that additional life years add additional opportunities to develop other NCDs.^14^ Contrary to some previous studies,^9,14,15^ this study found an association between male gender and TB-NCD multimorbidity. The higher prevalence of TB-NCD multimorbidity among men in this sample may be because alcohol and tobacco use disorders were included in the 10 NCDs assessed, stemming from the result that the two substance-use disorders were much more common in men than women in this study.

The likelihood of TB-NCD multimorbidity was higher in TB patients with lower education and poorer socio-economic status (poverty) than those with better socio-economic status, which is in agreement with previous studies and reviews.^14,15^ Contrary to an expected link between HIV-positive status and NCDs,^32^ this study found a negative association between HIV-positive status and NCD multimorbidity, whereas the Brazil study did not find any association.^9^ Compared to new TB patients, TB retreatment patients were more likely to have NCD multimorbidity. In TB retreatment, patients’ immune surveillance may be stronger compromised than in new TB patients emphasising the link between active TB disease and NCDs.^10^ This factor is important for the health care management process. Further, having a history of STI was associated with TB-NCD multimorbidity. This may be because STI is a clinically observed association with TB.^6^ The negative effects of TB-NCD multimorbidity on physical and mental health and quality of life, as found in previous studies,^16,17,18^ were confirmed in this study where the prevalence of physical health decreased, and common mental disorders and PTSD increased with an increase in the number of NCDs. This finding has significant health care implications for TB patients with multimorbidity in South Africa. It could mean that TB patients with multimorbidity would need more outpatient and inpatient care.^16^ In addition, primary health care service delivery will have to move away from a single chronic condition focused management to a more integrated primary care approach.^16^

### Study limitations

Caution should be taken when interpreting the results of this study because of certain limitations. The study was limited to only three selected health districts in South Africa, and the inclusion of more districts could have provided a different profile. As this was a cross-sectional study, causality between the compared variables cannot be concluded. A further limitation was that most variables, including NCDs and STIs (other than HIV), were assessed by self-report and desirable responses may have been given and NCDs under reported. Further, the poverty measure only assessed availability or non-availability of items in the past week instead of the past month. The study used a count of NCDs as a measure of multimorbidity, implying that each chronic condition has an equal impact on an individual, whereas in reality disease severity, the specific combination of NCDs and access to health care may affect multimorbidity.

## Conclusions

This is the first study in Africa to provide evidence of TB-NCD comorbidity and multimorbidity, its pattern and impact on health outcomes. The high prevalence of NCD multimorbidity found calls for an increased attention on the routine delivery of multimorbidity interventions. Older TB male patients of lower socio-economic status seem to be at the highest risk of multimorbidity, in particular in the presence of substance-use disorders.
